# Integrated Soil–Crop System Management Promotes Microbial Necromass and Soil Organic Carbon Sequestration in Black Soil Through Microbial Transformation and Aggregate Protection

**DOI:** 10.3390/microorganisms14102348

**Published:** 2026-10-14

**Authors:** Yantian Cui, Yan Zhou, Ranran Zhou, Jingmin Yang, Zhongqing Zhang, Jinhua Liu, Shuxia Liu, Yao Liang, Qi Li, Hongguang Cai

**Affiliations:** 1 College of Resource and Environmental Science, Jilin Agricultural University, Changchun 130118, China; 2 Institute of Agricultural Resources and Environment, Jilin Academy of Agricultural Sciences (Northeast Agricultural Research Center of China)/Key Laboratory of Black Soil Conservation and Utilization, Ministry of Agriculture and Rural Affairs, Changchun 130033, China; 3 Institute of Eco-Environment and Industrial Technology, Shanxi Agricultural University, Taiyuan 030031, China

**Keywords:** soil organic carbon, microbial necromass carbon, carbon use efficiency, microbial life-history strategies, aggregate protection

## Abstract

Long-term intensive cultivation has degraded soil structure and reduced soil organic carbon (SOC) retention in the black soils of Northeast China. However, the processes underlying SOC accumulation under integrated soil–crop system management (ISSM) remain poorly understood. An 11-year field experiment compared farmers’ practice (FP), high-yield and high-efficiency practice (IP), super-high-yield practice (HY), and ISSM. We assessed SOC stocks, aggregate stability, organic carbon chemistry, extracellular enzyme activities, microbial carbon-use traits, microbial community composition, and microbial necromass carbon (MNC), and used partial least squares path modeling as an exploratory analysis of potential linkages. Compared with FP, ISSM increased SOC content and stock by 87.0 and 77.0%, respectively, and achieved an SOC sequestration rate of 2.8 Mg C ha^−1^ yr^−1^. ISSM also increased mean weight diameter and geometric mean diameter by 45.0 and 61.0%, increased microbial biomass carbon and carbon use efficiency by 205.2 and 100.0%, and decreased qCO_2_ by 71.1%. Bacterial and fungal necromass carbon increased by 100.0 and 70.0%, respectively. The relative abundance of taxa operationally classified as growth-oriented also increased under ISSM. However, MNC/SOC did not differ significantly among treatments, while SOC aromaticity and hydrophobicity declined. Path analysis suggested that microbial carbon transformation was positively associated with necromass formation, whereas aggregate stability was associated with MNC and SOC retention. These results indicate that ISSM synchronously enlarged SOC and microbial necromass pools through sustained carbon inputs, efficient microbial transformation, and aggregate-mediated physical protection rather than through increased chemical recalcitrance.

## 1. Introduction

The black soil region of Northeast China is a major commodity grain production base and one of the country’s principal maize-producing areas, and its soil quality is therefore directly related to national food security [1]. However, long-term intensive cultivation and inappropriate nutrient inputs have increased crop yields, but these gains have been accompanied by soil structural degradation, reduced aggregate stability, and losses of soil organic carbon (SOC) [2,3]. SOC is fundamental to maintaining soil fertility, structural stability, and nutrient-supplying capacity, and it also constitutes an important component of the terrestrial ecosystem carbon pool [4]. Therefore, enhancing SOC sequestration in black soils through optimized farmland management while maintaining high crop productivity is an urgent scientific issue for black soil conservation and sustainable agricultural development [5].

The long-term persistence of SOC does not depend solely on the direct input and chemical recalcitrance of plant residues but is also regulated by microbial assimilation, transformation, and necromass formation [6]. Microorganisms convert plant-derived organic carbon into microbial biomass, which subsequently forms microbial necromass following cell death [7]. Microbial necromass can enter relatively stable soil carbon pools through mineral association and aggregate occlusion and is therefore regarded as an important source of stable SOC [8,9]. Bacterial and fungal necromass differ in their rates of formation, turnover characteristics, and stabilization pathways and may therefore contribute differently to carbon accumulation in agricultural soils [10]. Consequently, elucidating how different management practices affect the formation and preservation of bacterial and fungal necromass carbon is essential for understanding the mechanisms underlying SOC sequestration in croplands.

The accumulation of microbial necromass carbon depends on both its production and preservation [11]. Soil aggregates can physically protect microbial necromass by spatially restricting contact among microorganisms, extracellular enzymes, and organic matter [12]. Organic material inputs, tillage disturbance, and nutrient management can, in turn, influence the preservation efficiency of necromass carbon by altering aggregate formation and stability [13]. Meanwhile, soil carbon, nitrogen, and phosphorus availability regulates microbial investment in extracellular enzymes, carbon use efficiency, and respiratory carbon loss, thereby affecting microbial biomass production and necromass accumulation [14]. Changes in resource availability may also drive shifts in microbial communities between resource-conservative and fast-growing life-history strategies, consequently altering microbial carbon turnover and necromass formation [15]. Previous studies have generally examined aggregate protection, microbial metabolism, or community composition separately, and a systematic understanding of how these processes interact to regulate microbial necromass carbon sequestration remains lacking.

In recent years, integrated soil–crop system management (ISSM), centered on organic material inputs, reduced tillage, optimized planting density, and precision nutrient management, has been shown to substantially improve crop yield and soil fertility [16]. By coordinately optimizing planting density, straw return, organic and mineral fertilizer inputs, and tillage regimes, ISSM simultaneously considers crop productivity, resource-use efficiency, and soil quality [17]. Compared with individual management practices, ISSM may concurrently alter organic carbon inputs, aggregate structure, microbial resource limitation, and community composition, thereby affecting the formation and stabilization of microbial necromass carbon [18]. Previous studies have primarily focused on the effects of ISSM on crop yield, nutrient use, and SOC content. However, direct evidence from long-term field experiments remains limited regarding whether ISSM promotes microbial necromass carbon accumulation through the coupled effects of aggregate-mediated physical protection, microbial resource allocation, and community life-history strategies.

Accordingly, this study was conducted using an 11-year field experiment in the black soil region of Northeast China to compare the effects of farmers’ practice (FP), high-yield and high-efficiency practice (IP), super-high-yield practice (HY), and integrated soil–crop system management (ISSM) on SOC accumulation and structural characteristics, aggregate stability, microbial extracellular enzyme activities and nutrient limitation, microbial carbon-use characteristics, community composition, and microbial necromass carbon. The study aimed to elucidate the biotic and abiotic pathways through which integrated farmland management regulates microbial necromass carbon sequestration in black soil and to provide a theoretical basis for increasing SOC and promoting sustainable management in the black soil region of Northeast China. We hypothesized that: (i) Through sustained organic material inputs and coordinated nutrient and tillage management, ISSM would promote macroaggregate formation and improve aggregate stability, thereby enhancing the physical protection and retention of plant- and microbial-derived carbon; (ii) ISSM would improve microbial carbon utilization and biomass production by alleviating microbial carbon limitation and optimizing the allocation of extracellular enzymes; and (iii) ISSM would promote the accumulation of bacterial and fungal necromass carbon by altering microbial community composition and life-history strategies.

## 2. Materials and Methods

### 2.1. Experimental Site and Design

The field experiment was initiated in 2009 at the experimental station of the Jilin Academy of Agricultural Sciences in Gongzhuling City, Jilin Province, Northeast China (43°29′55″ N, 124°48′43″ E). The region has a temperate continental monsoon climate, with a mean annual precipitation of 595 mm, a mean annual temperature of 6.3 °C, and a frost-free period of 144 d. The soil was classified as a typical black soil according to the FAO soil classification system. The initial soil physicochemical properties were as follows: pH: 5.07, soil organic carbon: 16.30 g·kg^−1^, total nitrogen: 1.61 g·kg^−1^, available phosphorus: 7.23 mg·kg^−1^, available potassium: 151 mg·kg^−1^, and bulk density: 1.21 g·cm^−3^.

Four farmland management practices were established in the long-term field experiment: (1) farmers’ practice (FP), in which field management followed local farmers’ conventional cropping and production practices; (2) high-yield and high-efficiency practice (IP), in which crop yield was increased by 15–20% relative to local farmers’ practice through optimized crop management, including planting density, fertilization, and straw return; (3) super-high-yield practice (HY), a management system designed to increase crop yield by 30% without consideration of production inputs or environmental costs; and (4) integrated soil—crop system management (ISSM), a sustainable integrated management strategy developed on the basis of the super-high-yield system to increase crop yield and resource-use efficiency while reducing resource and environmental costs through the optimization of planting density, fertilization, and tillage practices. Detailed fertilization regimes and field management practices for each treatment are presented in Table S1.

The experiment was arranged in a randomized complete block design with 16 plots, comprising four treatments and four replicates. Each plot measured 6 m × 23 m. A single-season summer maize cropping system was used, with the maize cultivar Zhongdan 99 sown in late April and harvested in early October each year. The amount of straw returned to each plot corresponded to the maize residue remaining after harvest, comprising leaves and stems and accounting for 30% of the total dry matter. The organic fertilizer consisted of a thoroughly composted mixture of pig and cattle manure.

### 2.2. Soil Sampling

Following maize harvest in October 2020, five soil cores were randomly collected from the 0–20 cm layer of each plot using a soil auger with an inner diameter of 5 cm. The five soil cores from each plot were combined to form one composite sample, yielding a total of 16 composite samples. The samples were placed in sealed containers, transported to the laboratory in insulated boxes, and processed immediately. Stones, roots, and visible plant and animal residues were carefully removed from the fresh soil.

Each composite sample was immediately divided into four subsamples before drying. The first subsample was gently broken apart along natural planes, air-dried at room temperature, and used for the determination of soil physicochemical properties, SOC content, aggregate-size distribution, aggregate-associated SOC, amino sugars, and organic carbon chemical structure. The second subsample was stored at 4 °C and analyzed within 7 d for the determination of microbial biomass carbon, microbial respiration, and microbial carbon use efficiency using the ^18^O-H_2_O tracer method. The third subsample was stored at −20 °C and analyzed within 14 d for the measurement of extracellular enzyme activities. The fourth subsample was stored at −80 °C until DNA extraction, which was completed within one month after sampling, for microbial community analysis. All measurements based on fresh soil were corrected to an oven-dry soil mass basis according to the gravimetric soil moisture content.

### 2.3. Soil Analyses

#### 2.3.1. Determination of Soil Physicochemical Properties

Soil organic carbon (SOC) and total nitrogen (TN) were determined using an elemental analyzer (Vario MAX, Elementar Analysensysteme GmbH, Langenselbold, Germany). Soil available phosphorus (AP) was extracted with 0.5 mol·L^−1^ NaHCO_3_ and determined using the molybdenum-antimony colorimetric method. Soil available potassium (AK) was extracted with NH_4_OAc and quantified by flame photometry. Soil pH was measured using a pH electrode at a soil-to-water ratio of 1:2.5 (*w*/*v*). Soil bulk density was determined using the core method. Soil nitrate nitrogen (NO_3_^−^-N) and ammonium nitrogen (NH_4_^+^-N) were extracted with 0.01 mol·L^−1^ KCl and analyzed using an automated continuous-flow analyzer (TRAACS-2000, Bran+Luebbe, Norderstedt, Germany).

#### 2.3.2. Soil Organic Carbon Stock and Sequestration Rate

Soil organic carbon stock, the mean annual change in SOC, and the amount of SOC sequestered over the experimental period were calculated following the method of Huang et al. [19], using the following equations:

(1) $$\mathrm{SOC}\;\mathrm{stock}=\;\mathrm{SOC}\;\times \;\mathrm{BD}\;\times \frac{H}{100},$$

(2) $$\mathrm{MAC}=\frac{{\mathrm{SOC}}_{t}-{\mathrm{SOC}}_{0}}{t},$$

SOC_MS_ = BD × SOC_RR_ × H × 0.1, (3)

where BD is the soil bulk density (g·cm^−3^), and H is the soil sampling depth (0–20 cm in this study). MAC represents the mean annual change in SOC; t is the duration of the field experiment; SOC_0_ is the SOC content measured in the first year of the experiment; and SOC_t_ is the SOC content measured in the sampling year. SOCMS represents the mean annual SOC sequestration rate (Mg C ha^−1^ yr^−l^), and 0.1 is the unit conversion factor used in the equation.

#### 2.3.3. Soil Aggregate Fractionation

Soil water-stable aggregates were fractionated according to the method of Cambardella [20]. Before sieving, visible gravel, roots, and other impurities were removed from the soil samples to minimize interference with subsequent measurements. For each sample, 50 g of soil (<8 mm) was weighed and sequentially passed through sieves with mesh sizes of 2, 0.25, and 0.053 mm. After sieving, the aggregates retained on each sieve were rinsed into beakers with distilled water and dried at 60 °C to a constant weight. Three aggregate-size fractions were obtained: macroaggregates (2–0.25 mm), free microaggregates (0.25–0.053 mm), and non-aggregated silt plus clay (<0.053 mm). The mass proportion of each aggregate-size fraction was then calculated. The mean weight diameter (MWD), geometric mean diameter (GMD), and mass proportion of aggregates >0.25 mm (R_0.25_) were calculated using Equations (4) and (5).

(4) $$\mathrm{MWD}=\sum_{i=1}^{n}x_{i}\times w_{i},$$

(5) $$\mathrm{GMD}=\exp[\frac{\sum_{i=1}^{n}w_{i}\;\times \;{\mathrm{lnx}}_{i}}{\sum_{i=1}^{n}w_{i}}],$$

(6) $$R_{0.25}=\frac{M_{r>0.25}}{M_{T}},$$

where w_i_ is the mass percentage (%) of aggregates in size class i; x_i_ is the mean diameter of aggregate size class i; M_r_ ≥ 0.25 is the mass of aggregates ≥ 0.25 mm (g); and M_T_ is the total mass of aggregates (g).

#### 2.3.4. Determination of Chemical Structure of Organic Carbon

Functional groups of organic carbon were characterised by solid-state ^13^C NMR spectroscopy, following the method of Schmidt [21]. Soil samples were pretreated with a 10% HF solution to improve the signal-to-noise ratio of the obtained NMR spectra. The procedure was as follows: 5 g of air-dried soil was weighed into a 100 mL plastic centrifuge tube. 50 mL of 10% hydrofluoric acid solution was added, and the tube was shaken for 1 h, then centrifuged at 4500 rpm for 10 min. The supernatant was discarded, and the residue was further washed with the hydrofluoric acid solution. The process was repeated eight times. The oscillation times were 1 h × 4 times, 12 h × 3 times, and 24 h × 1 time. The treated samples were washed three times with distilled water to remove the residual hydrofluoric acid. Solid-state ^13^C cross-polarization magic angle spin (CPMAS) NMR spectra of the samples were recorded using a Bruker AVANCE III 400 WB spectrometer (Bruker AVANCE III 400 WB, Bruker Biospin, Karlsruhe, Germany). The parameters were as follows: resonance frequency 100.63 MHz; magic angle spin frequency 6 kHz; pulse delay time 0.5 s; and acquisition time 10 ms. Functional groups assignments followed Xiong et al. [22]: alkyl C (0–50 ppm), methoxyl C (50–60 ppm), O-alkyl C (60–95 ppm), di-O-alkyl C (95–110 ppm), aryl C (110–145 ppm), phenolic C (145–160 ppm), and carboxyl C (160–190 ppm).

The ratios of alkyl C to oxygenated alkyl C (A/O-A), aliphatic C to aromatic C (Alip/Arom), and hydrophobic C to hydrophilic C (HB/HI) were used as indicators for evaluating stability, aliphaticity, and hydrophobicity of the soil organic matter, respectively. They were calculated according to Equations (7)–(10) [23]:

(7) $$A/O-A=\frac{\mathrm{AlkylC}}{O-\mathrm{alkylC}},$$

(8) $$\mathrm{Alip}/\mathrm{Arom}=\frac{\mathrm{AlkylC}+O-\mathrm{alkylC}}{\mathrm{AromaticC}},$$

(9) $$\mathrm{HB}/\mathrm{HI}=\frac{\mathrm{AlkylC}+\mathrm{AromaticC}}{O-\mathrm{alkylc}+\mathrm{CarbonylC}},$$

Aromaticity = (AromaticC/AlkylC + O − AlkylC + AromaticC) × 100,(10)

#### 2.3.5. Soil Enzyme Activities and Microbial Nutrient Limitation

Soil extracellular enzyme activities were measured according to Saiya-Cork et al. [24]. Four hydrolytic enzymes associated with C, N, and P cycling were assayed: β-glucosidase (BG), β-N-acetylglucosaminidase (NAG), leucine aminopeptidase (LAP), and acid phosphatase (AP). Briefly, 1 g of soil was homogenized in 125 mL of 50 mmol sodium acetate buffer. Subsequently, 200 μL of the soil suspension and 50 μL of substrate solution were dispensed into each well of a black 96-well microplate. The microplates were incubated in the dark at 20 °C for 4 h. Enzyme activities were quantified by measuring fluorescence at excitation and emission wavelengths of 365 and 450 nm, respectively, using a Synergy H1 microplate reader (BioTek Instruments, Winooski, VT, USA). Details of the substrates are provided in Table S2.

The stoichiometry of soil enzyme activities was analyzed to determine potential microbial C, N, and P limitation. The vector characteristics of enzyme stoichiometry, including vector length and vector angle, were calculated using Equations (11) and (12) according to Moorhead et al. [25]. These values were used as indicators of microbial nutrient limitation: a greater vector length indicated stronger microbial C limitation, whereas the vector angle reflected the relative limitation by P versus N, with a larger angle indicating stronger P limitation [26].

(11) $$\mathrm{Vector}\;\mathrm{length}\;=\sqrt{\ln(\mathrm{BG})/{\ln(\mathrm{NAG}+\mathrm{LAP})}^{2}+{\left(\ln(\mathrm{BG})/\ln(\mathrm{AP})\right)}^{2}},$$

Vector angle = DEGREES { ATAN [ ln(BG)/ln(AP), ln(BG)/ln(NAG + LAP)]},(12)

#### 2.3.6. Determination and Calculation of Amino Sugars

Amino sugars were determined according to Appuhn et al. [27]. Briefly, soil samples were hydrolyzed with 6 mol·L^−1^ HCl at 105 °C for 8 h. The hydrolysates were adjusted to pH 6.6–6.8 and centrifuged at 1000 rpm for 10 min, after which the supernatants were collected and freeze-dried. The dried residues were redissolved in methanol to extract the amino sugars. Amino sugars were analyzed using an Agilent 6890A gas chromatograph equipped with a flame ionization detector (FID) and an HP-5 capillary column. Before purification, myo-inositol was added as an internal standard for quantifying amino sugar concentrations. N-Methylglucamine was used to assess recovery efficiency. Among the detected amino sugars, glucosamine (GlcN) and muramic acid (MurN) were selected to estimate the accumulation of fungal and bacterial necromass, respectively. The calculations were performed as follows:

BNC (Bacterial necromass carbon) = MurA × 45,(13)

FNC (Fungal necromass carbon) = (Glun/179.17 − 2 × MurA/251.23) × 179.17 × 9,(14)

MNC (Microorganism necromass carbon) = BNC + FNC,(15)

The FNC/BNC, BNC/SOC, FNC/SOC, and MNC/SOC ratios were subsequently calculated.

#### 2.3.7. Soil Microbial Carbon Use Efficiency

Microbial carbon use efficiency (CUE) was determined using the ^18^O-H_2_O tracer method, from which microbial growth rates were estimated [28]. Briefly, ^18^O-H_2_O (97.0 atom%) was added to screw-cap bottles containing soil samples to achieve a final ^18^O enrichment of 20.0 atom%. Control samples received an equal volume of unlabeled distilled water. All samples were incubated continuously at 25 °C for 24 h. Headspace gas was then sampled once, and CO_2_ concentrations were measured using a gas chromatograph (7890A GC, Agilent Technologies, Santa Clara, CA, USA). The soil samples were stored at −80 °C until DNA was extracted from both labeled and control soils using a FastDNA^TM^ SPIN Kit for Soil. DNA concentrations were determined by UV-visible spectrophotometry using a NanoDrop instrument (NanoDrop Technologies Inc., Wilmington, DE, USA). The ^18^O abundance and oxygen content of the extracted DNA were measured using an isotope-ratio mass spectrometer coupled to a thermal conversion elemental analyzer (IRMS–TC/EA; Thermo Fisher Scientific, Bremen, Germany). Total microbial growth or the microbial growth rate (C_growth_) was expressed as microbial biomass carbon production per unit time. DNA production was converted to MBC production based on the linear regression relationship between the increase in soil DNA and MBC concentration during incubation. Microbial respiration was estimated from the amount of CO_2_-C released during incubation, representing respiratory carbon release or the respiration rate (C_respiration_). Under the steady-state assumption, total microbial carbon uptake or the carbon uptake rate (C_uptake_) was calculated as the sum of C_growth_ and C_respiration_. Microbial carbon use efficiency and microbial biomass turnover time were calculated as follows:

(16) $$\mathrm{CUE}\left(\mathrm{Microbial carbon utilization efficiency}\right)=\frac{C_{\mathrm{growth}}}{C_{\mathrm{growth}}+C_{\mathrm{respiration}}},$$

#### 2.3.8. Determination of Microbial Quotient and Metabolic Quotient

Soil microbial biomass carbon (MBC) was determined using the chloroform fumigation–extraction method described by Vance et al. [29]. Soil microbial respiration was measured after a 14 d pre-incubation using a method modified from Anderson and Domsch [30]. Briefly, 20 g of fresh soil was placed in an incubation bottle, and soil moisture was adjusted to 60% of field water-holding capacity. The samples were aerobically pre-incubated at 25 °C for 14 d. On day 14, the bottles were sealed for 7 h, after which the headspace CO_2_ concentration was measured using an EGM-5 portable infrared gas analyzer (PP Systems, Amesbury, MA, USA). The amount of CO_2_-C released during the 7 h closure period was calculated according to the change in headspace CO_2_ concentration, effective headspace volume, incubation temperature, closure duration, and oven-dry equivalent mass of the soil. Microbial respiration (MR) was expressed as μg CO_2_–C g^−1^ dry soil h^−1^.

The microbial quotient (Cmic/Corg) and microbial metabolic quotient (q_CO2_) were calculated based on MBC, SOC, and microbial respiration using the following equations:

(17) $$\mathrm{Cmic}/\mathrm{Corg}=\frac{\mathrm{MBC}}{\mathrm{SOC}},$$

(18) $$q_{\mathrm{co}2}=\frac{\mathrm{MR}}{\mathrm{MBC}}$$

where MBC represents microbial biomass carbon, SOC represents soil organic carbon, and MR represents the CO_2_–C production rate measured during the 7 h closure period following the 14 d pre-incubation. Cmic/Corg represents the proportion of microbial biomass carbon relative to soil organic carbon, reflecting the microbial biomass contribution to the soil carbon pool. q_CO2_ represents microbial metabolic quotient, calculated as microbial respiration per unit microbial biomass carbon, indicating the metabolic efficiency and maintenance cost of soil microbial communities.

#### 2.3.9. Determination of Soil Microbial Community Composition

Total soil microbial DNA was extracted from 0.5 g of fresh soil using a FastDNA^®^ SPIN Kit for Soil (MP Biomedicals, Illkirch, France) according to the manufacturer’s instructions. DNA integrity was assessed by 1.2% agarose gel electrophoresis. The V3–V4 region of the bacterial 16S rRNA gene was amplified using the primer pair 338F (5′-ACTCCTACGGGAGGCAGCAG-3′) and 806R (5′-GGACTACHVGGGTWTCTAAT-3′), whereas the fungal ITS1 region was amplified using the primer pair ITS1F (5′-CTTGGTCATTTAGAGGAAGTAA-3′) and ITS2R (5′-GCTGCGTTCTTCATCGATGC-3′). PCR amplification was performed with an initial denaturation at 95 °C for 3 min, followed by 30 cycles of denaturation at 95 °C for 30 s, annealing at 55 °C for 30 s, and extension at 72 °C for 45 s, with a final extension at 72 °C for 10 min. The PCR products were recovered from 2% agarose gels, purified using an AxyPrep DNA Gel Extraction Kit (Axygen Biosciences, Union City, CA, USA), and quantified using QuantiFluor™-ST (Promega, Madison, WI, USA). The purified amplicons were sequenced on an Illumina MiSeq platform by Shanghai Personalbio Biotechnology Co., Ltd. (Shanghai, China).

Raw paired-end sequence reads were demultiplexed, primer-trimmed, merged, and quality-filtered using QIIME 2 version 2025.7 [31]. Low-quality reads, reads containing ambiguous bases, and sequences outside the expected length range were removed. Chimeric sequences were identified and discarded using VSEARCH version 2.29.4. The remaining high-quality sequences were dereplicated and clustered into operational taxonomic units (OTUs) at 97.0% sequence identity using VSEARCH. Representative bacterial and fungal OTU sequences were taxonomically assigned against the Greengenes database, release 13.8, and the UNITE database, release 8.0, respectively. Chloroplast, mitochondrial, non-target, and singleton OTUs were removed, and the resulting OTU tables were rarefied to an equal sequencing depth before downstream analyses.

Based on previously reported ecological tendencies, Alphaproteobacteria, Actinomycetota, Acidobacteriota, Gemmatimonadota, and Gammaproteobacteria were operationally assigned to the K-associated bacterial group, whereas Bacteroidota, Bacillota, Chloroflexota, and Verrucomicrobiota were assigned to the r-associated bacterial group. Ascomycota and Basidiomycota were operationally classified as r- and K-associated fungal groups, respectively [15,32]. These assignments were used to construct community-level life-history indices and do not imply that all members within each higher taxonomic group share identical life-history traits.

### 2.4. Statistical Analysis

All statistical analyses were performed using SPSS 21.0 and R version 4.5.0. Differences among the four farmland management treatments were assessed using one-way analysis of variance (ANOVA). Statistical significance was determined at *p* < 0.05. Data are presented as means ± standard errors based on four field replicates (*n* = 4).

Pearson correlation analysis was used to examine the relationships between microbial necromass carbon and soil physicochemical properties, aggregate stability, extracellular enzyme activities, microbial carbon-related traits, and microbial community composition. Simple linear regression was further performed to evaluate the relationships between the relative abundances of dominant bacterial and fungal taxa and bacterial, fungal, and total microbial necromass carbon. Regression coefficients, coefficients of determination (R^2^), *p* values, and 95% confidence intervals were reported.

Partial least squares path modeling (PLS-PM) was conducted in R to evaluate the direct and indirect pathways through which farmland management affected the soil carbon pool via soil environmental conditions, microbial life-history strategy, extracellular enzyme activity, microbial carbon transformation, microbial necromass carbon, and aggregate-mediated physical protection. Variables were standardized before model construction. The significance of standardized path coefficients was assessed using bootstrap resampling, and the explanatory power of each endogenous variable was evaluated using R^2^. Model performance was additionally described using the goodness-of-fit index. Additionally, the Mnova software (9.0.0) was used to process the data, the Origin 2021 software and R 4.5.0 was employed for graphing.

## 3. Results

### 3.1. Effects of Different Field Management Practices on Soil Organic Carbon Stocks and Sequestration Rates

**Figure 1:**
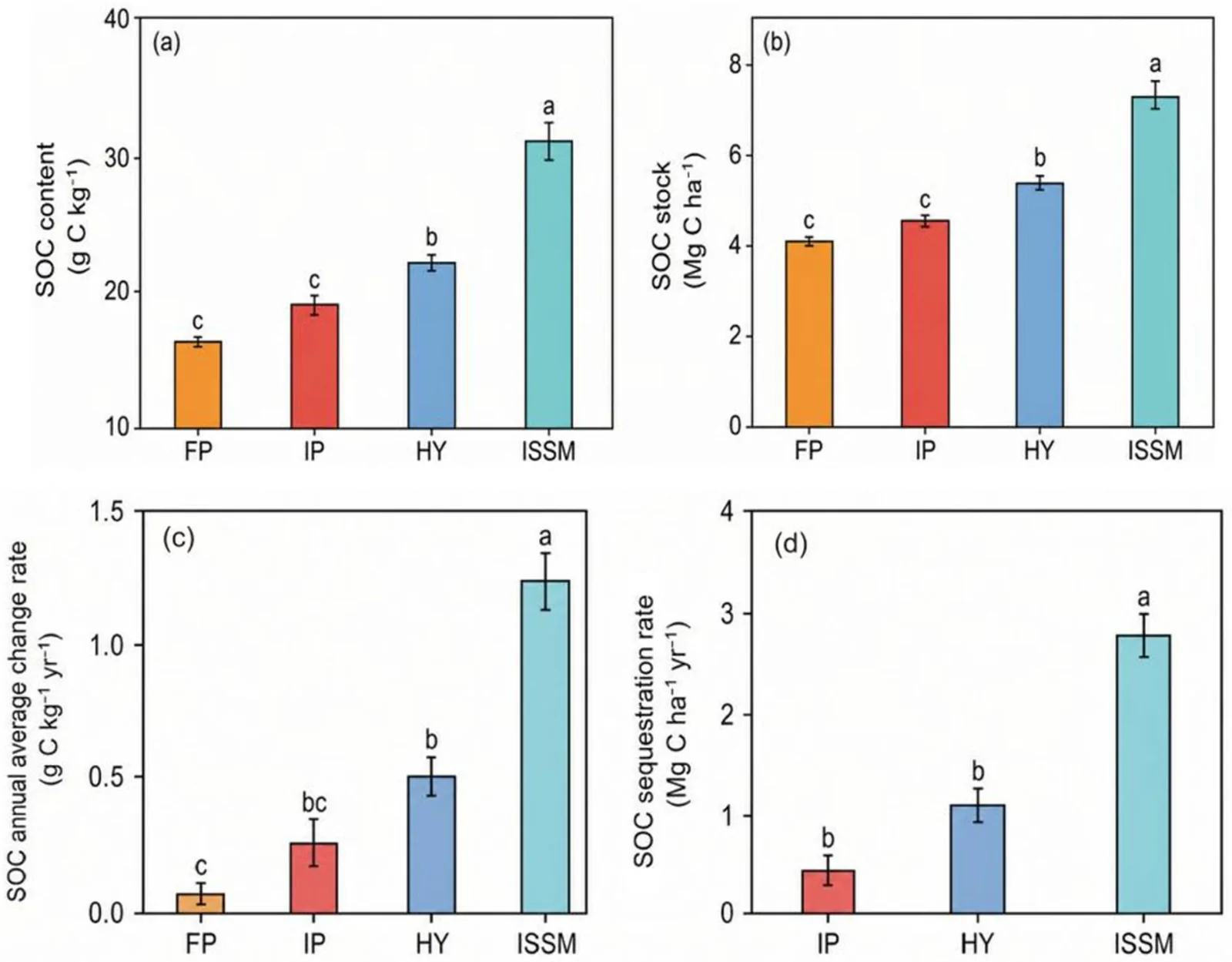
Effects of different farmland management practices on soil organic carbon content, stock, and accumulation rates. (**a**) Soil organic carbon (SOC) content; (**b**) SOC stock; (**c**) annual average change rate of SOC content; and (**d**) SOC sequestration rate relative to farmer practice. FP, farmer practice; IP, improved high-yield and high-efficiency practice; HY, super-high-yield practice; ISSM, integrated soil–crop system management. Bars represent means ± standard errors (*n* = 4). Different lowercase letters above the bars within the same panel indicate significant differences among treatments at *p* < 0.05.

Long-term farmland management significantly affected soil organic carbon (SOC) content and stock (Figure 1a,b). Compared with FP, the other three treatments increased SOC content by 15.0–87.0% and SOC stock by 11.0–77.0%. Both variables were significantly greater under ISSM than under the other treatments (*p* < 0.05). HY also resulted in significantly greater SOC content and stock than FP and IP, whereas IP and FP did not differ significantly.

The mean annual change in SOC content ranged from 0.03 to 1.25 g C kg^−1^ yr^−1^ (Figure 1c). The rate under ISSM differed significantly from those under the other treatments. HY showed a significantly greater rate than FP, whereas IP did not differ significantly from either FP or HY. Using FP as the reference, the concentration-based SOC sequestration rates under IP, HY, and ISSM were 0.2, 0.5, and 1.2 g C kg^−1^ yr^−1^, respectively. The corresponding area-based SOC sequestration rates were 0.5, 1.1, and 2.8 Mg C ha^−1^ yr^−1^ (Figure 1d). ISSM differed significantly from IP and HY, whereas no significant difference was detected between IP and HY.

### 3.2. Effects of Different Field Management Practices on Soil Organic Carbon Stability

**Figure 2:**
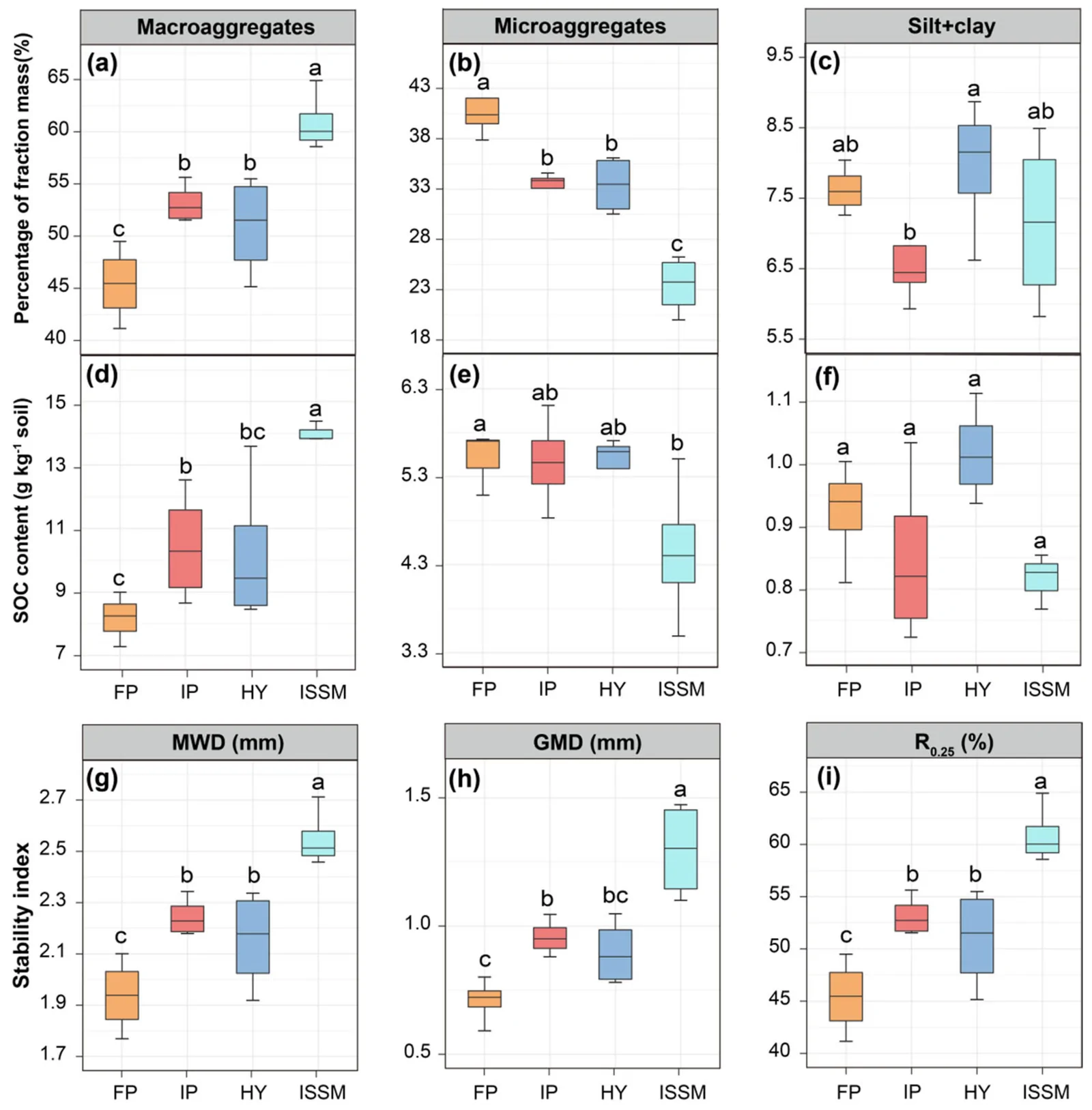
Effects of different farmland management practices on soil aggregate-size distribution, aggregate-associated soil organic carbon, and aggregate stability. (**a**) Mass proportions of macroaggregates (2–0.25 mm); (**b**) microaggregates (0.25–0.053 mm); (**c**) silt plus clay (<0.053 mm); (**d**–**f**) soil organic carbon (SOC) contents in the corresponding aggregate-size fractions; (**g**) the mean weight diameter (MWD); (**h**) geometric mean diameter (GMD); and (**i**) proportion of aggregates > 0.25 mm (R_0.25_) under farmers’ practice (FP), high-yield and high-efficiency practice (IP), super-high-yield practice (HY), and integrated soil–crop system management (ISSM). Different lowercase letters indicate significant differences among treatments at *p* < 0.05. Boxes represent the interquartile range, horizontal lines within boxes indicate medians, and whiskers represent the ranges of the observations.

The distribution of soil aggregate-size fractions under the different field management practices is shown in Figure 2a–c. Compared with FP, the proportions of macroaggregates > 250 μm were significantly increased by 17.0, 11.0, and 34.0% under IP, HY, and ISSM, respectively (*p* < 0.05), with the greatest increase observed under ISSM. In contrast, the proportion of microaggregates (0.25–0.053 mm) decreased significantly under all improved management practices, by an average of 19, 18, and 43.0% under IP, HY, and ISSM, respectively, relative to FP. No significant differences in the <0.053 mm fraction were observed among the four management practices.

The effects of different farmland management practices on aggregate-associated soil organic carbon contents are shown in Figure 2d–f. Compared with FP, ISSM significantly increased the organic carbon contents of all aggregate-size fractions except the <0.053 mm fraction, which remained unchanged (*p* < 0.05). The organic carbon contents of the 2–0.25 mm and 0.25–0.053 mm aggregate fractions were significantly higher than those of the <0.053 mm fractions (*p* < 0.05). The mean organic carbon contents of the 2–0.25 mm, 0.25–0.053 mm, and <0.053 mm aggregate fractions were 18.9, 16.5, and 12.6 g·kg^−1^, respectively. Across the four farmland management practices, aggregate-associated organic carbon contents was highest in the 2–0.25 mm fractions.

As shown in Figure 2g–i, all improved management practices (IP, HY, and ISSM) significantly enhanced soil aggregate stability compared with conventional FP. The mean weight diameter (MWD) increased by 16 and 45% under IP and ISSM, respectively, whereas the geometric mean diameter (GMD) increased by 23 and 61%, respectively (*p* < 0.05). The proportion of macroaggregates > 0.25 mm (R_0.25_) showed a similar pattern, increasing by 17.0 and 34.0% under IP and ISSM, respectively. Although IP and HY exhibited comparable MWD and R_0.25_ values, no significant differences in GMD were detected among FP, IP, and HY. Collectively, these results indicate that ISSM produced the greatest improvement in soil aggregate characteristics.

**Figure 3:**
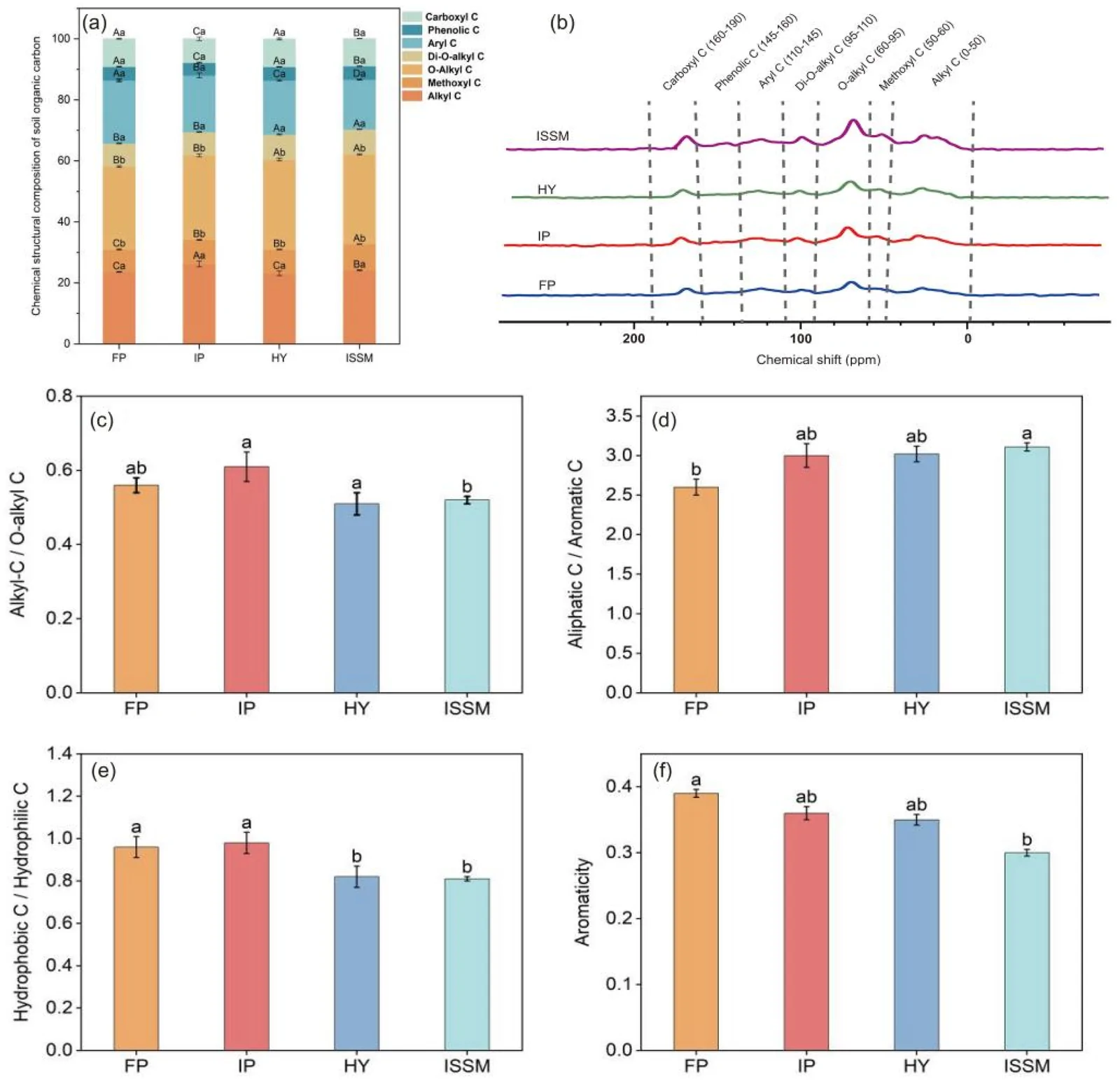
Effects of different farmland management practices on the chemical structure and stability of soil organic carbon. (**a**) Relative proportions of the major organic carbon functional groups; (**b**) solid-state ^13^C nuclear magnetic resonance (^13^C NMR) spectra; (**c**) alkyl C/O-alkyl C ratio; (**d**) aliphatic C/aromatic C ratio; (**e**) hydrophobic C/hydrophilic C ratio; and (**f**) aromaticity under farmers’ practice (FP), high-yield and high-efficiency practice (IP), super-high-yield practice (HY), and integrated soil–crop system management (ISSM). The functional groups included alkyl C, methoxyl C, O-alkyl C, di-O-alkyl C, aryl C, phenolic C, and carboxyl C. Different lowercase letters indicate significant differences among treatments at *p* < 0.05.

The chemical structure of soil organic carbon under the different field management practices is shown in Figure 3a. Across all treatments, O-alkyl C accounted for the largest proportion (27.2–29.5%), followed by Alkyl C (23.1–26.2%), Aryl C (16.3–20.7%), and Carboxyl C (7.85–9.17%). Compared with FP, HY increased the proportion of O-alkyl C by 8.3% and decreased that of Aryl C by 14.1%. Under ISSM, the proportions of methoxyl C and O-alkyl C increased by 17.2 and 8.1%, respectively, whereas that of aryl C decreased by 21.3% (*p* < 0.05). However, no significant changes were observed in di-O-alkyl C, Carboxyl C, Alkyl C, or phenolic C. Overall, HY and ISSM increased the proportion of labile organic carbon while decreasing that of recalcitrant organic carbon.

The chemical stability indices of soil organic carbon under the different field management practices are shown in Figure 3c–f. No significant differences in the alkyl C/O-alkyl C ratio were observed among FP, IP, and HY. Compared with FP, ISSM slightly decreased the alkyl C/O-alkyl C ratio, although the difference was not significant. The aliphatic C/aromatic C ratio was highest under ISSM among the four management practices and was significantly increased by 30% relative to FP, but did not differ significantly from those under IP and HY. Compared with FP, the hydrophobic C/hydrophilic C ratio decreased by 14.7 and 12.5% under HY and ISSM, respectively. Compared with IP, it decreased by 15.5 and 13.3% under HY and ISSM, respectively, whereas no significant difference was observed between FP and IP. Aromaticity was highest under FP and lowest under ISSM, while no significant difference was detected between IP and HY.

### 3.3. Effects of Different Farmland Management Practices on Soil Microbial Necromass Carbon Accumulation

**Figure 4:**
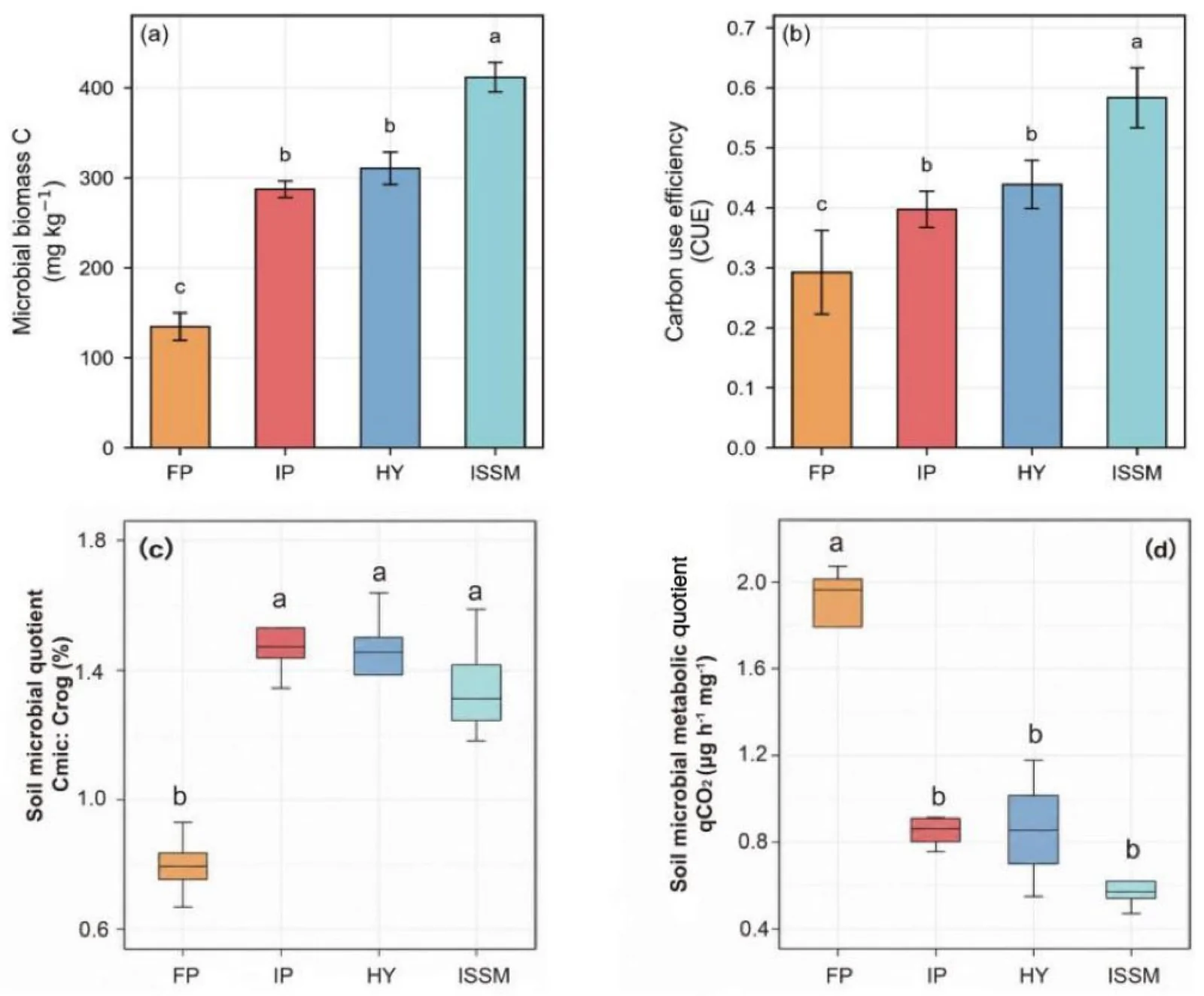
Effects of different farmland management practices on soil microbial carbon-related properties. (**a**) Soil microbial biomass carbon (MBC); (**b**) microbial carbon use efficiency (CUE); (**c**) microbial quotient (Cmic/Corg); and (**d**) microbial metabolic quotient (qCO_2_). Data in (**a**,**b**) are presented as means ± SE (*n* = 4). In (**c**,**d**), the central lines represent medians, the boxes represent interquartile ranges, and the whiskers represent the data ranges (*n* = 4). Different lowercase letters indicate significant differences among treatments (*p* < 0.05). FP, farmers’ practice; IP, high-yield and high-efficiency practice; HY, super-high-yield practice; ISSM, integrated soil–crop system management.

Farmland management practices significantly affected soil microbial biomass carbon (MBC) and microbial carbon use efficiency (CUE) (Figure 4a,b). Compared with FP, MBC increased by 112.6, 129.6, and 205.2% under IP, HY, and ISSM, respectively, whereas CUE increased by 37.9, 51.7, and 100.0%, respectively. Both MBC and CUE were significantly higher under IP and HY than under FP, with no significant difference between IP and HY. ISSM resulted in significantly higher MBC and CUE than all other treatments (*p* < 0.05).

The microbial quotient (Cmic/Corg) and microbial metabolic quotient (qCO_2_) exhibited contrasting responses to farmland management (Figure 4c,d). Compared with FP, Cmic/Corg increased by 83.8, 81.3, and 65.0% under IP, HY, and ISSM, respectively, whereas qCO_2_ decreased by 56.3, 56.9, and 71.1%, respectively. Cmic/Corg was significantly higher and qCO_2_ was significantly lower under IP, HY, and ISSM than under FP, with no significant differences among the three optimized management treatments.

**Figure 5:**
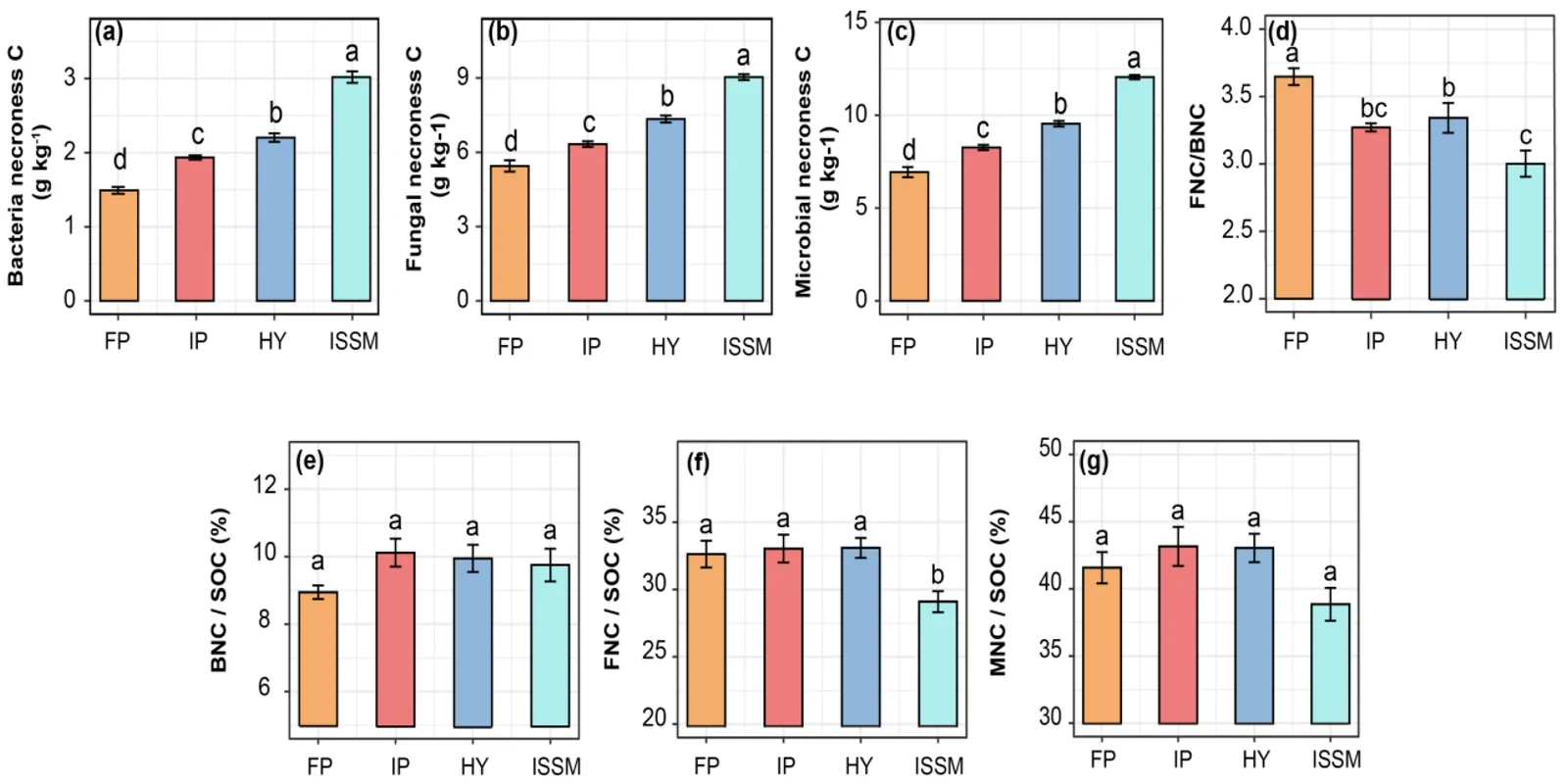
Effects of different farmland management practices on soil microbial necromass carbon accumulation and its contribution to soil organic carbon. (**a**) Bacterial necromass carbon (BNC); (**b**) fungal necromass carbon (FNC); (**c**) total microbial necromass carbon (MNC); (**d**) the FNC/BNC ratio; (**e**) the BNC/SOC ratio; (**f**) the FNC/SOC ratio; and (**g**) the MNC/SOC ratio under farmers’ practice (FP), high-yield and high-efficiency practice (IP), super-high-yield practice (HY), and integrated soil–crop system management (ISSM). Different lowercase letters above the bars indicate significant differences among treatments at *p* < 0.05.

The responses of microbial necromass carbon (MNC) to different farmland management practices are shown in Figure 5. Compared with FP, IP, and HY, ISSM resulted in the greatest accumulation of MNC, with bacterial necromass carbon (BNC), fungal necromass carbon (FNC), and total MNC increasing by 40–100, 20–70, and 30–70%, respectively (Figure 5a–c). Notably, the FNC/BNC ratio differed significantly among management practices (Figure 5d). The ratio was highest under FP and lowest under ISSM, whereas IP and HY showed intermediate values with no significant difference between them.

Analysis of necromass distribution patterns showed that BNC accounted for 8.9–10.1% of SOC across all treatments, whereas FNC accounted for 29.0–33.0% of SOC. The contribution of microbial necromass to SOC was lowest under ISSM, decreasing by 10.8, 11.9, and 12.0% relative to FP, IP, and HY, respectively. However, the proportional contribution of microbial necromass to SOC did not differ significantly among the management practices.

### 3.4. Effects of Different Field Management Practices on Soil Nutrient Limitation and Microbial Life-History Strategies

**Figure 6:**
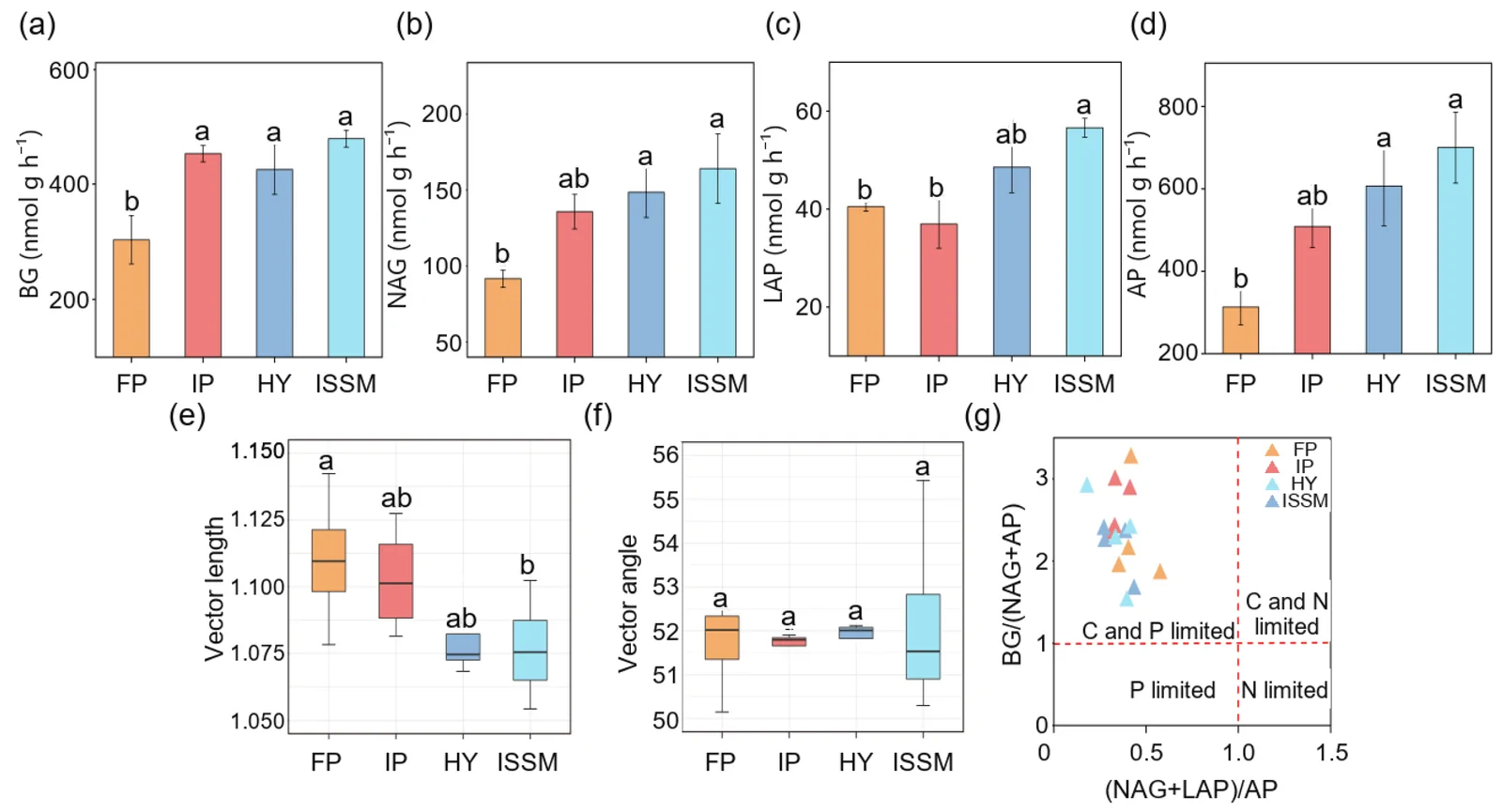
Effects of different farmland management practices on soil extracellular enzyme activities and microbial nutrient limitation. (**a**) Activities of β-glucosidase (BG); (**b**) activities of β-N-acetylglucosaminidase (NAG); (**c**) activities of leucine aminopeptidase (LAP); (**d**) activities of acid phosphatase (AP); (**e**) enzyme stoichiosmetric vector length; (**f**) enzyme stoichiosmetricvector angle (**f**); and (**g**) ecoenzymatic stoichiometry-based microbial nutrient limitation under farmers’ practice (FP), high-yield and high-efficiency practice (IP), super-high-yield practice (HY), and integrated soil–crop system management (ISSM). Different lowercase letters indicate significant differences among treatments at *p* < 0.05. In panel (**g**), dashed lines separate the regions representing C and P co-limitation, C and N co-limitation, P limitation, and N limitation.

Changes in soil extracellular enzyme activities under the different farmland management practices are shown in Figure 6. Compared with FP, IP, HY, and ISSM significantly increased the activities of enzymes associated with C, N, and P cycling. β-Glucosidase activity increased by 40.0–58.0% under IP, HY, and ISSM relative to FP, with the highest activity observed under ISSM. Similarly, β-N-acetylglucosaminidase activity increased by 62.0 and 79.0% under HY and ISSM, respectively, whereas no significant difference was observed between IP and FP. Notably, leucine aminopeptidase activity under ISSM was significantly higher than that under FP and IP, by 40 and 53%, respectively. Acid phosphatase activity exhibited a pattern similar to that of β-N-acetylglucosaminidase, with significant differences between HY or ISSM and FP. Compared with FP, acid phosphatase activity increased by 1.9- and 2.2-fold under HY and ISSM, respectively.

The enzyme stoichiometry-based analysis of nutrient limitation revealed significant differences among the four management practices (Figure 6e–g), following the order FP > IP > HY > ISSM. The greater vector length under FP indicated stronger microbial C limitation, whereas the reduced vector length under ISSM indicated alleviation of C limitation. Vector angles exceeded 45° under all treatments, suggesting potential P limitation relative to N limitation. However, no significant differences in vector angle were observed among treatments.

**Figure 7:**
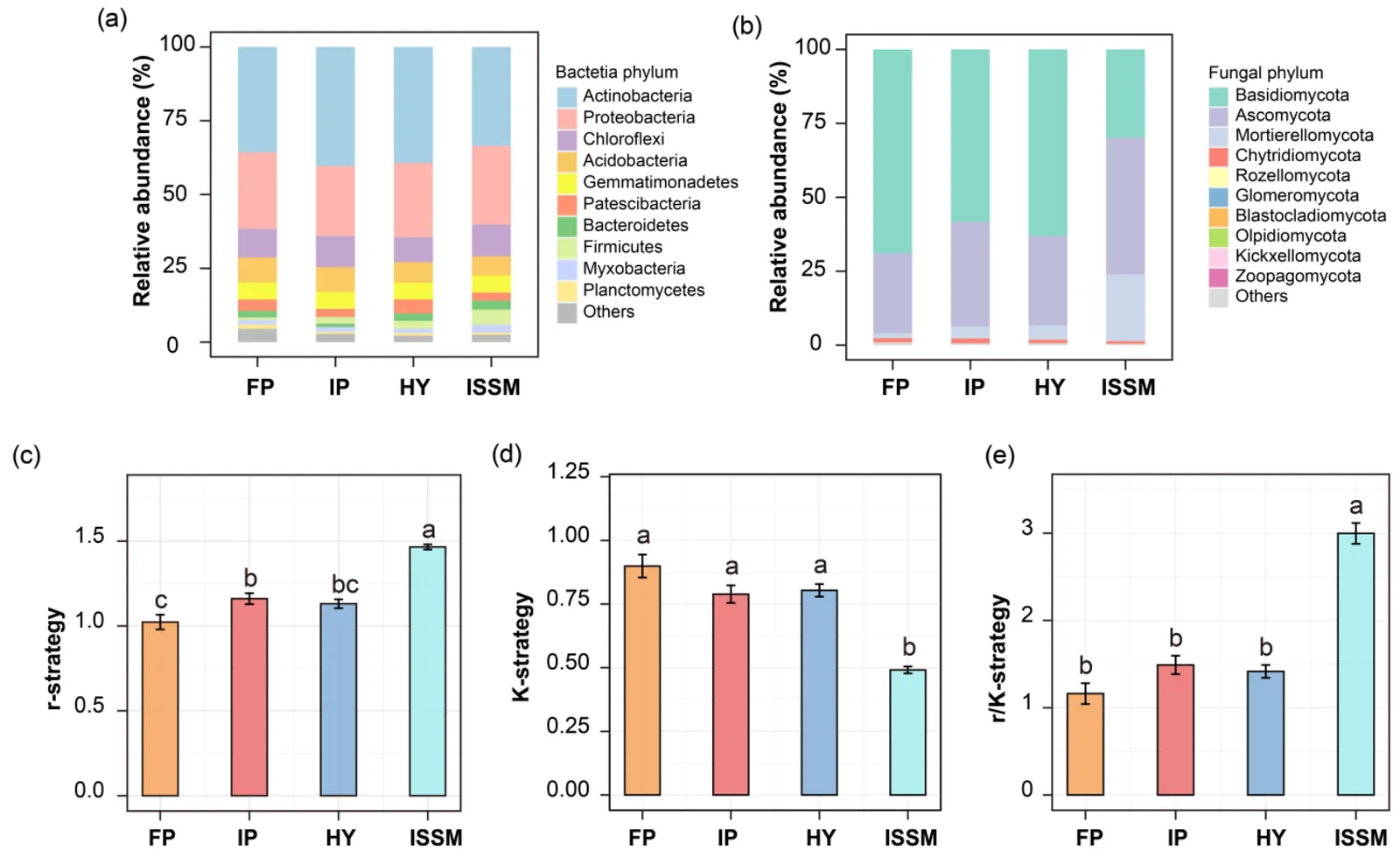
Effects of different farmland management practices on soil microbial community composition and life-history strategies. (**a**) Relative abundances of the dominant bacterial; (**b**) relative abundances of the dominant fungal; (**c**) relative abundances of r-strategists; (**d**) relative abundances of K-strategists; and (**e**) the r/K ratio. FP, farmer practice; IP, improved high-yield and high-efficiency practice; HY, super-high-yield practice; ISSM, integrated soil–crop system management. Different lowercase letters above the bars indicate significant differences among treatments at *p* < 0.05.

As shown in Figure 7, the different field management practices significantly affected soil microbial community structure and drove shifts in microbial life-history strategies. Across all treatments, the bacterial communities were dominated by Actinobacteriota (34.0–40.0%), Proteobacteria (24.0–27.0%), Chloroflexi (8.0–11.0%), and Acidobacteriota (7.0–8.0%). However, the management practices exerted significant selective effects on the microbial communities. Compared with conventional farmers’ practice (FP), both the super-high-yield practice (HY) and integrated soil–crop system management (ISSM) significantly enriched Firmicutes, whose relative abundance increased by 2.9- and 5.6-fold, respectively. In addition, Bacteroidetes and Myxococcota reached their highest relative abundances under ISSM, indicating that ISSM promoted the proliferation of copiotrophic microorganisms by improving carbon availability. In contrast, the oligotrophic phylum Acidobacteriota exhibited the opposite trend, with its relative abundance decreasing by 1, 2, and 2% under IP, HY, and ISSM, respectively, compared with FP. The fungal communities across all treatments were dominated by Basidiomycota (30.0–69.0%), Ascomycota (27.0–46.0%), Mortierellomycota (1.6–23.0%), and Chytridiomycota (0.8–1.4%). Notably, ISSM markedly altered fungal community composition. Compared with FP, the relative abundances of Ascomycota and Mortierellomycota under ISSM increased by 70.0% and 14-fold, respectively. In contrast, the relative abundance of Basidiomycota decreased by 57.0% under ISSM, and that of Glomeromycota also decreased significantly.

Based on the ecological characteristics of the dominant taxa, the ten most abundant bacterial and fungal phyla were classified as r-strategists (fast-growing taxa) or K-strategists (resource-conservative taxa) (Figure 7c,d). The results showed that the different management practices induced distinct microbial life-history strategies. ISSM significantly enriched r-strategists, whose abundance was 1.3–1.4-fold higher than that under the other treatments, whereas FP maintained a greater proportion of K-strategists, which was 1.1–1.8-fold higher than that under IP, HY, and ISSM. Importantly, the r/K ratio followed a consistent pattern across all treatments: ISSM > IP > HY > FP (*p* < 0.05). These results indicate that ISSM shifted the microbial community from a resource-conservative strategy toward a fast-growing strategy by increasing soil resource availability and imposing moderate physical disturbance.

### 3.5. Mechanisms Underlying the Effects of Different Field Management Practices on Microbial Necromass Carbon Sequestration

**Figure 8:**
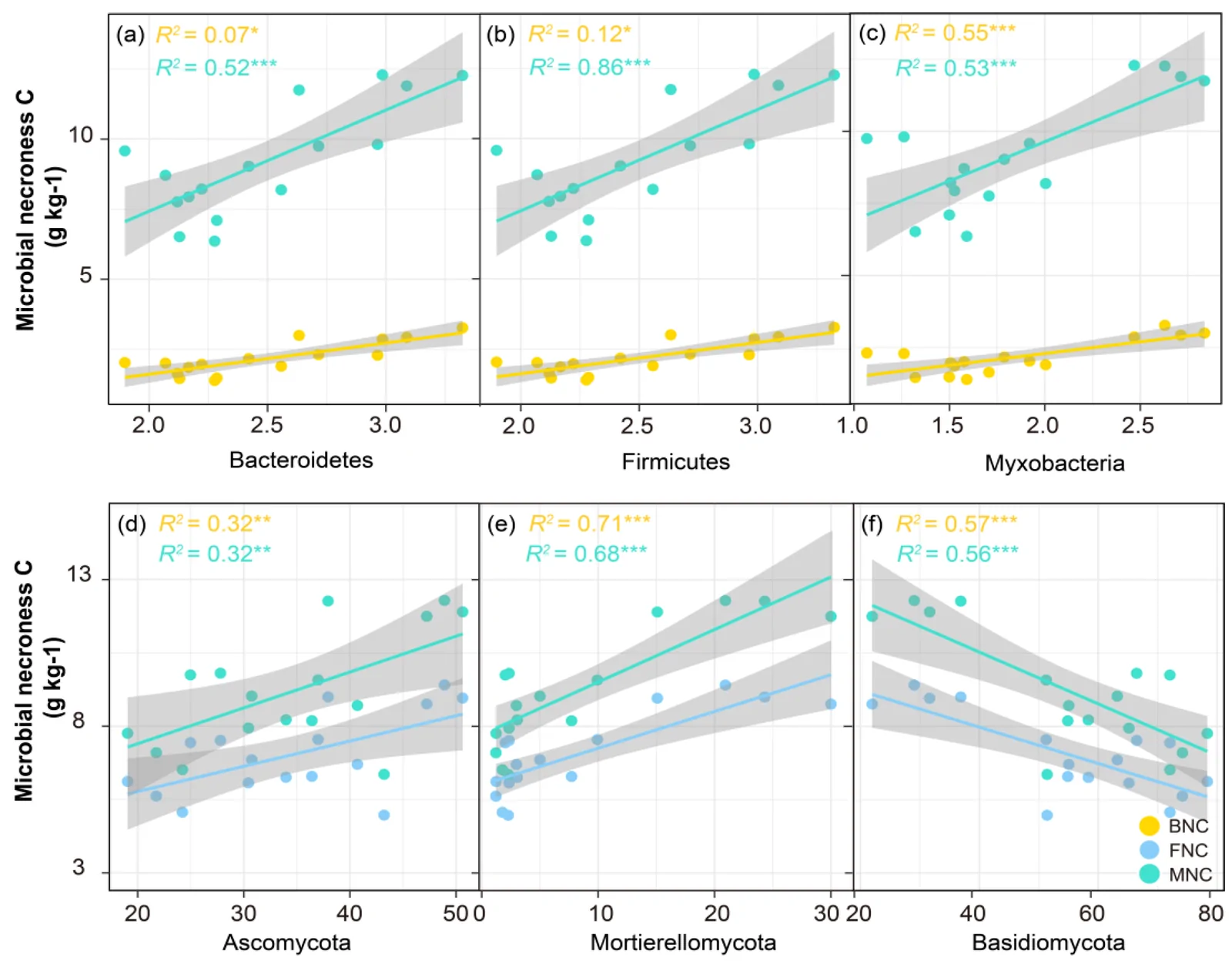
Relationships between the relative abundances of different microbial taxa and microbial necromass carbon contents. (**a**) Bacteroidetes, (**b**) Firmicutes, (**c**) Myxobacteria, (**d**) Ascomycota, (**e**) Mortierellomycota, and (**f**) Basidiomycota. Yellow, light-blue, and turquoise points and regression lines represent bacterial necromass carbon (BNC), fungal necromass carbon (FNC), and total microbial necromass carbon (MNC), respectively. Solid lines indicate linear regression fits, and gray shaded areas represent 95% confidence intervals. The coefficients of determination (R^2^) are shown in each panel. *, **, and *** indicate significance at *p* < 0.05, *p* < 0.01, and *p* < 0.001, respectively.

Linear regression analysis revealed distinct relationships between dominant microbial taxa and microbial necromass carbon (Figure 8). Among bacterial taxa, Bacteroidetes and Firmicutes were positively associated with bacterial necromass carbon (BNC), although they explained relatively little of its variation (R^2^ = 0.07 and 0.12, respectively). Their relationships with total microbial necromass carbon (MNC) were stronger, particularly for Firmicutes (R^2^ = 0.86), followed by Bacteroidetes (R^2^ = 0.52). Myxobacteria was positively correlated with both BNC and MNC, explaining 55.0 and 53.0% of their variation, respectively.

Among fungal taxa, Ascomycota was positively associated with fungal necromass carbon (FNC) and MNC, with both relationships explaining 32.0% of the observed variation. Mortierellomycota showed the strongest positive relationships with FNC and MNC (R^2^ = 0.71 and 0.68, respectively). In contrast, Basidiomycota was negatively associated with both FNC and MNC, explaining 57.0 and 56.0% of their variation, respectively. Collectively, these results indicate that the enrichment of Firmicutes, Myxobacteria, Ascomycota, and Mortierellomycota was closely associated with increased microbial necromass carbon accumulation, whereas the relative abundance of Basidiomycota exhibited the opposite pattern.

**Figure 9:**
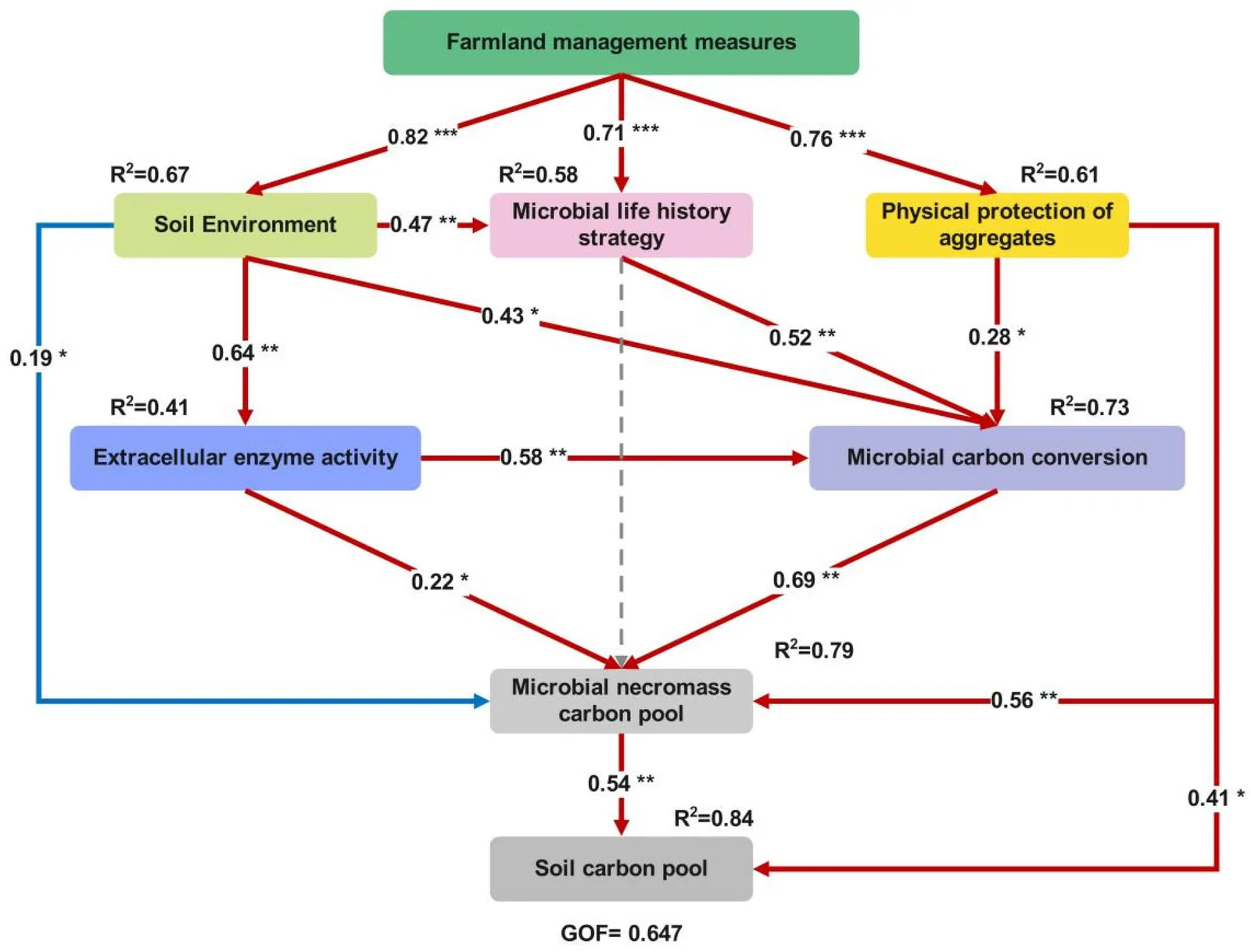
Partial least squares path model showing the effects of different farmland management practices on the microbial necromass carbon and soil organic carbon pools in black soil. The partial least squares path model (PLS-PM) illustrates the direct and indirect pathways through which farmland management practices affect the microbial necromass carbon pool and soil organic carbon pool via the soil environment, microbial life-history strategies, extracellular enzyme activities, microbial carbon transformation, and aggregate-mediated physical protection. Values adjacent to the arrows are standardized path coefficients. Red and blue solid arrows indicate significant positive and negative effects, respectively, whereas gray dashed arrows indicate nonsignificant effects. Arrow widths are proportional to the absolute values of the path coefficients. The R^2^ values adjacent to the endogenous variables represent the proportions of variance explained by the model. The overall goodness-of-fit index (GOF) was 0.647. *, **, and *** indicate significance at *p* < 0.05, *p* < 0.01, and *p* < 0.001, respectively.

The PLS-PM results showed that farmland management synergistically promoted soil organic carbon accumulation by improving the soil environment, regulating microbial life-history strategies, and strengthening aggregate-mediated physical protection (Figure 9). Improvements in the soil environment enhanced extracellular enzyme activities and microbial carbon transformation efficiency, whereas microbial life-history strategies and aggregate protection also significantly promoted carbon transformation. Microbial carbon transformation was the primary driver of necromass carbon formation (β = 0.69), and the necromass carbon pool subsequently significantly increased the soil carbon pool (β = 0.54). Meanwhile, aggregate-mediated physical protection promoted necromass carbon preservation and directly enhanced soil carbon retention. The model explained 79.0% and 84.0% of the variation in the organic carbon pool and the total soil carbon pool in black soil, respectively, indicating that microbial-mediated carbon sequestration processes and the physical protection provided by aggregates within the ISSM system are key factors driving soil carbon accumulation.

## 4. Discussion

Soil organic carbon (SOC) sequestration in black soil is strongly mediated by microbial transformation and is sensitive to agricultural management, whereas the persistence of newly formed SOC depends on microbial necromass production and its subsequent protection within aggregates and mineral-associated fractions [7,9,10]. Based on an 11-year field experiment in the black soil region of Northeast China, this study systematically compared the effects of farmers’ practice, high-yield and high-efficiency practice, super-high-yield practice, and integrated soil–crop system management on SOC accumulation, microbial carbon metabolism, community life-history strategies, microbial necromass formation, and aggregate stability. The results showed that ISSM did not increase SOC solely through increased exogenous organic carbon inputs. Instead, it simultaneously enlarged the SOC and microbial necromass carbon pools by improving soil resource availability, enhancing microbial carbon transformation efficiency, and strengthening aggregate-mediated physical protection. Compared with FP, ISSM increased SOC content and stock by 87.0 and 77.0%, respectively, and significantly increased bacterial, fungal, and total microbial necromass carbon. However, the proportion of MNC in SOC did not differ significantly among treatments, whereas the aromaticity and hydrophobicity of organic carbon decreased under ISSM. These findings indicate that SOC accumulation under ISSM was not primarily attributable to increased chemical recalcitrance but rather to the coordinated effects of sustained carbon inputs, efficient microbial transformation, and aggregate-mediated physical protection.

### 4.1. Synergistic Enhancement of SOC and MNC Under ISSM

Exogenous organic matter inputs provide the material basis for SOC accumulation in croplands, but their net contribution to SOC sequestration depends on microbial assimilation and transformation, respiratory carbon losses, and subsequent stabilization within aggregate-occluded and mineral-associated carbon pools [33,34,35]. Compared with FP, IP, and HY, ISSM produced the highest SOC content, stock, and sequestration rate, suggesting that the integrated implementation of straw return, organic fertilizer application, optimized nutrient management, and tillage regulation was more effective in promoting organic carbon retention than management strategies emphasizing yield enhancement or high external inputs alone. Continuous organic material inputs not only directly supplied plant-derived carbon but may also have increased belowground carbon inputs through greater root residue production and rhizodeposition associated with enhanced crop productivity [36,37]. The SOC sequestration rate under ISSM reached 2.8 Mg C ha^−1^ yr^−1^, demonstrating substantial net SOC accumulation over the 11-year experimental period and suggesting that cumulative carbon gains exceeded cumulative carbon losses at the system level.

Meanwhile, ISSM significantly increased the absolute contents of BNC, FNC, and MNC. The greater increase in BNC than in FNC resulted in a lower FNC/BNC ratio. This pattern may reflect enhanced bacterial biomass production and turnover under resource-enriched conditions, leading to greater bacterial necromass formation through repeated microbial growth and death. Previous global evidence indicates that cropland soils generally have a higher bacterial-to-fungal necromass ratio and faster microbial biomass turnover than less intensively managed ecosystems [6]. Nevertheless, fungi may continue to contribute relatively persistent carbon through the decomposition of complex substrates, hyphal-mediated aggregate formation, and the slower degradation of fungal cell-wall residues [13]. This finding is consistent with the soil microbial carbon pump framework, whereby plant-derived organic carbon is assimilated and transformed into microbial biomass and metabolic products, with a proportion subsequently entering relatively persistent SOC pools as microbial necromass following cell death [7,38]. However, BNC/SOC, FNC/SOC, and MNC/SOC did not differ significantly among treatments, indicating that ISSM did not promote SOC accumulation by increasing the proportional contribution of microbial necromass carbon. Instead, it simultaneously expanded the pools of plant-derived carbon, microbial biomass, microbial necromass, and total SOC. Therefore, when interpreted together with the increases in MBC and CUE, the greater absolute amount of microbial necromass carbon provides stronger evidence that ISSM enhanced microbial carbon processing and necromass formation than the MNC/SOC ratio alone.

Changes in the chemical structure of organic carbon further clarified the mechanism underlying SOC accumulation under ISSM. Compared with FP, ISSM increased the proportions of methoxyl C and O-alkyl C and decreased aryl C, aromaticity, and hydrophobicity, whereas the alkyl C/O-alkyl C ratio did not increase significantly. These results indicate that the newly accumulated SOC did not exhibit greater overall chemical resistance to decomposition. The increase in O-alkyl C may have originated from the carbohydrate-rich constituents of straw, organic fertilizer, and root residues, whereas the decrease in aromaticity likely reflected the dilution of pre-existing SOC by fresh organic carbon inputs. According to the microbial efficiency–mineral stabilization framework, labile substrates do not necessarily lead to SOC loss because they can be rapidly assimilated by microorganisms and transformed into necromass and extracellular products, which are subsequently preserved through mineral association or aggregate occlusion [39]. Therefore, the simultaneous increase in SOC and decrease in chemical recalcitrance observed in this study are not contradictory. Rather, they indicate that SOC stabilization under ISSM depended primarily on sustained carbon inputs, microbial processing, and biophysical protection rather than on the selective accumulation of aromatic compounds [40].

### 4.2. Alleviation of Resource Limitation and Shifts in Life-History Strategies Promote Efficient Microbial Carbon Transformation

The capacity of microorganisms to convert exogenous carbon into biomass and necromass depends on the coordination of substrate supply, extracellular enzyme production, respiratory carbon losses, and microbial life-history strategies. ISSM significantly increased the activities of BG, NAG, LAP, and AP while decreasing the vector length of extracellular enzyme stoichiometry. These results indicate that ISSM enhanced the potential for extracellular depolymerization of C-, N-, and P-containing organic compounds while alleviating relative microbial C limitation [41,42]. Higher extracellular enzyme activities may have accelerated the decomposition of complex organic compounds in straw and organic fertilizer, thereby increasing the supply of low-molecular-weight carbon compounds and nutrients available for microbial assimilation [34]. In contrast, the greater vector length, lower CUE, and higher qCO_2_ under FP collectively suggest stronger microbial C limitation and a greater allocation of assimilated carbon to respiration and maintenance rather than biomass production. By continuously supplying organic substrates and improving nutrient availability, ISSM reduced the relative constraint imposed by C acquisition and allowed a greater proportion of assimilated carbon to be allocated to microbial growth, consistent with its higher CUE [14]. Enzyme vector angles exceeded 45° under all treatments and did not differ significantly among them, indicating potential microbial P limitation relative to N limitation across the experimental soil, with no detectable change in its magnitude among management practices. Therefore, the improvement in microbial carbon transformation efficiency under ISSM was primarily associated with alleviated C limitation and improved overall resource availability rather than with a marked change in relative P limitation.

The coordinated changes in MBC, CUE, Cmic/Corg, and qCO_2_ provide complementary evidence of altered microbial carbon cycling (Figure 5). Compared with FP, ISSM increased MBC by 205.2%, CUE by 100.0%, and Cmic/Corg by 65.0%, while decreasing qCO_2_ by 71.1%. Relative to FP, IP and HY also increased Cmic/Corg by 83.8% and 81.3% and decreased qCO_2_ by 56.3% and 56.9%, respectively. However, neither quotient differed significantly among IP, HY, and ISSM. Cmic/Corg represents microbial biomass relative to SOC, whereas qCO_2_ reflects respiratory carbon loss per unit microbial biomass [43]. Their contrasting responses therefore indicate a greater microbial biomass share of SOC and lower biomass-specific respiration than under FP. Because MBC is the numerator of Cmic/Corg and the denominator of qCO_2_, these opposing trends alone do not establish a direct physiological relationship. Notably, ISSM had the greatest absolute MBC and CUE, but its Cmic/Corg was not significantly higher than those under IP and HY. This pattern suggests that microbial biomass expanded alongside the SOC pool rather than accounting for a disproportionately greater share of it. Under stronger resource limitation, more assimilated carbon may be required for substrate acquisition, extracellular enzyme production, cellular maintenance, and respiration, thereby constraining biomass production. Improved carbon and nutrient availability under ISSM may instead have favored microbial growth, consistent with its higher CUE and lower qCO_2_ relative to FP. Long-term field evidence likewise indicates that organic amendments can enhance microbial CUE, although the response depends on amendment quality and resource stoichiometry [44]. Together, the increases in MBC and CUE suggest a larger potential precursor supply for necromass formation.

Improved resource conditions also reshaped microbial community life-history strategies. ISSM significantly enriched Firmicutes, Bacteroidetes, Myxococcota, Ascomycota, and Mortierellomycota, increased the relative abundance of r-strategists and the r/K ratio, and reduced the relative dominance of resource-conservative taxa. These results indicate that the resource-rich environment created by long-term organic material inputs favored taxa characterized by rapid responses, fast growth, and high turnover. Bacteroidetes and Firmicutes can generally utilize fresh plant-derived carbon rapidly, whereas Mortierellomycota and some Ascomycota respond strongly to rhizosphere carbon and labile organic matter inputs [45]. Correlation analysis showed that these taxa were significantly and positively associated with BNC, FNC, or MNC, whereas Basidiomycota was negatively associated with FNC and MNC, further indicating that ISSM-induced shifts in community composition were consistent with changes in necromass carbon accumulation.

However, enrichment of fast-growing taxa does not necessarily result in SOC accumulation. Under insufficient substrate supply, stoichiometric imbalance, or excessive disturbance, rapid microbial activity may increase respiration and SOC mineralization. Therefore, the effect of growth-oriented microbial strategies on SOC depends on whether assimilated carbon is efficiently converted into biomass and subsequently preserved as microbial products [46]. ISSM not only increased the relative abundance of operationally defined r-strategists and the r/K ratio but also enhanced CUE and reduced qCO_2_, thereby favoring carbon allocation to microbial growth rather than respiratory loss. The PLS-PM results further showed that microbial life-history strategies affected MNC mainly indirectly through microbial carbon transformation, whereas microbial carbon transformation exerted a strong direct effect on MNC, with a path coefficient of 0.69. This indicates that the contribution of community restructuring to necromass accumulation was primarily expressed through changes in microbial physiological functioning rather than taxonomic composition alone. Thus, the pathway of “alleviation of resource limitation-shift toward growth-oriented strategies–efficient microbial carbon transformation-necromass formation” provides an integrated explanation for the microbial carbon pump under ISSM. No single microbial phylum should therefore be regarded as the sole driver of SOC accumulation.

### 4.3. Synergistic Effects of Efficient Microbial Transformation and Aggregate-Mediated Physical Protection on SOC Sequestration

An increase in microbial necromass does not necessarily ensure its long-term preservation. Newly formed microbial-derived carbon is more likely to enter relatively persistent SOC pools when protected through aggregate occlusion or mineral association. In this study, ISSM increased the proportion of macroaggregates > 250 μm by 34.0%, increased MWD and GMD by 45.0 and 61.0%, respectively, and significantly increased R_0.25_, while decreasing the proportion of microaggregates. This shift should not be interpreted simply as microaggregate loss; rather, it may reflect the incorporation of microaggregates and silt–clay particles into larger hierarchical aggregates, because microaggregates can serve as structural building units within macroaggregates [47]. Organic inputs from straw, organic fertilizer, and roots can provide particulate and dissolved organic binding materials that promote macroaggregate formation [48]. Meanwhile, microbial extracellular polymeric substances, fungal hyphae, and root-derived compounds can connect soil particles and enhance aggregate stability. The coordinated optimization of tillage and nutrient inputs under ISSM may also have reduced mechanical disturbance and favored aggregate persistence [49]. Therefore, ISSM likely strengthened the physical protection of microbial necromass by promoting hierarchical aggregation and reducing its accessibility to decomposers and extracellular enzymes.

Improved aggregate stability can reduce contact among microorganisms, extracellular enzymes, and organic substrates through spatial isolation, thereby suppressing the decomposition of newly formed necromass. Necromass and its metabolic products within aggregates may also associate further with clay minerals and metal ions, prolonging their residence time in soil [50]. In this study, MNC was significantly and positively correlated with MWD, GMD, and R_0.25_, and FNC was more strongly associated with aggregate stability. These relationships suggest that fungal hyphae and cell-wall residues may contribute simultaneously to aggregate formation and stable carbon accumulation. Fungal hyphae can physically entangle soil particles, whereas chitin, proteins, and amino sugar components in fungal necromass can be readily adsorbed onto mineral surfaces or stabilized through cation bridging. Consequently, fungal-derived carbon may be more readily preserved within aggregates than some plant-derived carbon. These findings are consistent with those of Zhao et al. [51], who reported that aggregate occlusion reduces the subsequent decomposition of microbial necromass.

The PLS-PM further showed that aggregate-mediated physical protection exerted positive effects on both the microbial necromass carbon pool and the total soil carbon pool. The model explained 79 and 84% of the variation in MNC and SOC. Higher CUE and MBC were associated with greater microbial biomass production and a larger potential supply of necromass, whereas improved aggregate stability increased the likelihood that newly formed microbial-derived carbon would be retained. Without sufficient physical or mineral protection, newly produced necromass may be rapidly reutilized by microorganisms or mineralized. Conversely, enhanced aggregate stability alone would have a limited effect on SOC accumulation without sustained inputs of plant- and microbial-derived carbon. Recent evidence similarly indicates that persistent SOC formation depends on the joint regulation of carbon inputs, microbial processing, aggregation, and mineral protection rather than on any single process [52,53]. Therefore, ISSM established an integrated sequestration pathway of “organic carbon input–efficient microbial transformation–necromass formation–aggregate occlusion and mineral association,” thereby coupling microbial carbon production with its subsequent preservation.

This coupled mechanism helps explain an important finding of the present study: although ISSM increased extracellular enzyme activities and enriched fast-growing microbial taxa, it promoted the simultaneous accumulation of SOC and MNC rather than accelerating net SOC loss. Sustained organic carbon inputs likely maintained substrate availability, while higher CUE favored the allocation of assimilated carbon to microbial biomass production, thereby expanding the potential precursor pool for necromass formation. However, CUE alone cannot ensure SOC accumulation because the resulting microbial products must subsequently be protected from rapid reutilization and mineralization [54]. Under ISSM, improved aggregate stability likely reduced the accessibility of newly formed microbial-derived carbon, while interactions with soil minerals may have provided additional physicochemical protection [55]. Thus, SOC accumulation under ISSM was not primarily attributable to the progressive formation of highly aromatic and chemically recalcitrant compounds. Instead, it reflected a coupled biological–physical stabilization process involving sustained carbon inputs, efficient microbial transformation, necromass formation, and subsequent aggregate and mineral protection. This interpretation is consistent with long-term evidence showing that organic inputs can promote SOC accumulation through the simultaneous expansion of plant-derived particulate carbon and microbially mediated, necromass-associated carbon pools [56].

Beyond documenting an increase in SOC stock, this study shows that the absolute pools of SOC and microbial necromass carbon can expand without a significant increase in the MNC/SOC ratio or SOC aromaticity. The concurrent changes in microbial carbon-use indicators and aggregate stability provide a framework for examining how microbial processing and physical protection may contribute to carbon retention in black soil. From a practical perspective, the 11-year field implementation of ISSM demonstrates that an integrated package of straw return, organic amendments, and coordinated nutrient, planting, and tillage management can be applied in the studied maize production system. However, to further promote this cultivation system, it is necessary to tailor it to local conditions and conduct site-specific and economic assessments, thereby achieving a synergistic improvement in agricultural resource utilization and economic returns.

## 5. Conclusions

Intensive cultivation has reduced soil organic carbon (SOC) in the black soils of Northeast China, yet it remains unclear how integrated soil–crop system management (ISSM) affects the size and stabilization of SOC and microbial necromass carbon (MNC) pools. To address this gap, we compared four management regimes in an 11-year replicated maize field experiment and quantified SOC stocks, MNC, microbial physiological traits, aggregate characteristics, and SOC chemical composition. Relative to farmers’ practice, ISSM increased SOC stock by 77% and achieved an SOC sequestration rate of 2.8 Mg C ha^−1^ yr^−1^. The absolute MNC pool, microbial biomass carbon, and carbon use efficiency also increased, whereas the microbial metabolic quotient declined. ISSM further improved aggregate stability, providing conditions potentially favorable to the retention of microbial-derived carbon. However, the MNC/SOC ratio did not differ significantly among treatments, and SOC aromaticity decreased. Thus, the principal advance of this study is to show that the expansion of SOC and MNC pools can occur without a greater proportional contribution of necromass or increased chemical recalcitrance. The combined observations and exploratory path analysis are consistent with complementary roles for microbial carbon processing and aggregate-mediated physical protection. In Northeast China, the tested combination of straw return, organic amendments, and coordinated nutrient, planting-density, and tillage management offers a practical field-tested framework for increasing carbon storage in black-soil maize systems. Elsewhere, these principles should be adapted to local soils, climate, organic-resource supply, crop requirements, and implementation costs, with long-term trials assessing both carbon gains and agricultural performance before broad adoption.

## Data Availability

The data presented in this study are available on request from the corresponding author.

## Supplementary Materials

The following supporting information can be downloaded at: https://www.mdpi.com/article/10.3390/microorganisms14102348/s1, Table S1: The detailed field management information for four farmland management practices; Table S2: Soil physicochemical properties in different farmland management practices.
