# Supplementary material for: Integrated Soil–Crop System Management Promotes Microbial Necromass and Soil Organic Carbon Sequestration in Black Soil Through Microbial Transformation and Aggregate Protection
Source: Microorganisms. 2026 Oct 14;14(10):2348. doi: 10.3390/microorganisms14102348 (PMC13707608; doi:10.3390/microorganisms14102348)
Supplement: Supplementary file 1 [file microorganisms-14-02348-s001.zip › microorganisms-4594101-supplementary.pdf]

Supplementary Table S1 The detailed field management information for four farmland management practices

| Treatment | N (kg· $ha^{-1}$) | $P_2O_5$ (kg· $ha^{-1}$) | $K_2O$ (kg· $ha^{-1}$) | organic fertilizer (kg· $ha^{-1}$) | straw returning | planting density (Plant/hectare) |
|---|---|---|---|---|---|---|
| FP | 300 | 120 | 120 | - | 30% | 50,000 |
| IP | 195 | 90 | 90 | - | 30% | 60,000 |
| HY | 300 | 120 | 120 | - | 30% | 70,000 |
| ISSM | 195 | 90 | 90 | 10000 | 30% | 70,000 |

Supplementary Table S2 Soil physicochemical properties in different farmland management practices

| **Treatment** | **pH** | **TN ($g·kg^{-1}$)** | **C/N** | **AP ($mg·kg^{-1}$)** | **AK($mg·kg^{-1}$)** | **$NO_3^-$-N($mg·kg^{-1}$)** | **$NH_4^+$-N($mg·kg^{-1}$)** | **BD($g·cm^3$)** |
|---|---|---|---|---|---|---|---|---|
| FP | 5.04 ± 0.07 b | 1.58 ± 0.10 b | 10.6 ± 0.46 a | 85.7 ± 3.08 b | 223 ± 13.5 c | 3.57 ± 0.39 b | 6.32 ± 0.49 bc | 1.23 ± 0.00 a |
| IP | 5.27 ± 0.22 ab | 1.69 ± 0.03 b | 11.4 ± 0.31 a | 84.6 ± 3.76 b | 241 ± 16.1 bc | 14.4 ± 0.59 a | 8.94 ± 0.44 a | 1.21 ± 0.03 a |
| HY | 5.02 ± 0.12 b | 1.96 ± 0.06 b | 11.4 ± 0.31 a | 105 ± 3.99 b | 309 ± 8.35 b | 11.1 ± 1.28 a | 7.79 ± 0.38 ab | 1.18 ± 0.01 a |
| ISSM | 5.70 ± 0.10 a | 2.87 ± 0.24 a | 11.0 ± 0.59 a | 175 ± 20.7 a | 457 ± 18.8 a | 14.8 ± 0.69 a | 5.22 ± 0.55 c | 1.16 ± 0.01 a |
